# Leaf to Root: Harnessing leaf spectral signatures for non-destructive monitoring of soybean nodule traits

**DOI:** 10.1016/j.plaphe.2026.100203

**Published:** 2026-03-24

**Authors:** K.H. Cheng, Kejing Fan, Xiewang Gao, Liping Wang, Hui Zhang, Feng Zhang, Fuk-Ling Wong, Zhihui Wang, Jin Wu, Shichao Jin, Hon-Ming Lam

**Affiliations:** a School of Life Sciences, The Chinese University of Hong Kong, Hong Kong Special Administrative Region of China; b Center for Soybean Research of the State Key Laboratory of Agrobiotechnology, The Chinese University of Hong Kong, Hong Kong Special Administrative Region of China; c Guangdong Provincial Key Laboratory of Remote Sensing and Geographical Information System, Guangdong Open Laboratory of Geospatial Information Technology and Application, Guangzhou Institute of Geography, Guangdong Academy of Sciences, Guangzhou, China; d School of Biological Sciences, The University of Hong Kong, Hong Kong Special Administrative Region of China; e Institute for Climate and Carbon Neutrality, The University of Hong Kong, Hong Kong Special Administrative Region of China; f State Key Laboratory of Crop Genetics and Germplasm Enhancement, Zhongshan Biological Breeding Laboratory, Collaborative Innovation Centre for Modern Crop Production co-sponsored by Province and Ministry, Jiangsu Key Laboratory of Soybean Biotechnology and Intelligent Breeding, Engineering Research Center of Plant Phenotyping, Ministry of Education, Academy for Advanced Interdisciplinary Studies, Nanjing Agricultural University, Nanjing, 211800, China; g Institute of Environment, Energy and Sustainability, The Chinese University of Hong Kong, Hong Kong Special Administrative Region of China

**Keywords:** Soybean, Nodule number and weight, Leaf spectra, Spectra-nodule trait associations, Precision agriculture

## Abstract

Soybean (*Glycine max*) root nodules, formed through symbiosis with nitrogen-fixing rhizobia, are essential for biological nitrogen fixation. While quantifying key nodulation traits, nodule number and weight, is critical for assessing symbiotic efficiency and yield potential, current methods are destructive and labor-intensive, unsuitable for longitudinal monitoring and high-throughput phenotyping. Here, we established hyperspectral leaf reflectance as a non-destructive, high-resolution tool capable of monitoring root nodule development. Using Partial Least Squares Regression models, we connected spectral data with nodule metrics from 528 unique soybean plants across 18 genotypes, inoculated with different rhizobium strains, and under different abiotic stresses. These models achieved high accuracy for predicting nodule number (R^2^ = 0.75, nRMSE = 6.02%) and moderate accuracy for nodule weight (R^2^ = 0.53, nRMSE = 12.38%). Crucially, spectral analyses revealed distinct hyperspectral signatures sensitive to nodule traits. While different rhizobium strains induced comparable changes in both nodule traits, and therefore produced highly overlapped spectral domains, diagnostically distinct spectral patterns were generated under drought versus salt stress, with the former suppressing nodulation more significantly than the latter. Furthermore, we demonstrated the effectiveness of our models for real-time *in-situ* monitoring of nodule development for individual plants. Spectral-nodule trait covariation analyses further revealed leaf signatures correlated with nodule traits primarily through systemic physiological coupling governed by carbon-nitrogen exchange dynamics and plant water status. This study showcased hyperspectral sensing as a transformative methodology, enabling the unprecedented non-destructive quantification of nodulation dynamics, revealing novel physiological insights into plant-microbe-environment interactions, facilitating breeding and management strategies for sustainable soybean production.

## 1 Introduction

Soybean (*Glycine max*) establishes a critical symbiotic relationship with nitrogen-fixing rhizobia, resulting in the formation of specialized root organs termed nodules [1]. Within these nodules, bacteroids convert atmospheric nitrogen (N_2_) into ammonia (NH_3_), providing the plant with essential biologically available nitrogen (N). This symbiosis substantially diminishes agricultural reliance on synthetic N fertilizers, yielding significant economic and environmental benefits [2]. Consequently, nodule traits, including number, weight, and nitrogen fixation activity, serve as fundamental indicators of symbiotic efficiency, directly influencing soybean biomass accumulation and grain yield [[3], [4], [5]]. Recent research showed that optimizing nodule number, while preventing energy-dissipating supernodulation, represents a precise strategy for enhancing yield sustainably [5]. However, nodule formation and function exhibit high sensitivity to environmental perturbations. Abiotic stresses, particularly drought and salinity, pose major global constraints to soybean production and profoundly disrupt the nodulation process [[6], [7], [8], [9]]. These stresses significantly reduce nodule number, weight, and nitrogenase activity [8,10], with salinity often impacting nodule number more severely than total nodule weight [10]. Crucially, distinct abiotic stresses can induce characteristic alterations in nodule traits even within the same genotype. Therefore, understanding these stress-specific responses is paramount for developing targeted mitigation strategies. Robust, effective and non-destructive methods for quantifying nodule traits across varying stress conditions are hereby essential to advance the fundamental understanding of stress-nodulation interactions and enable precision agriculture approaches.

Current methods of assessing nodule traits rely predominantly on destructive harvesting, which involves meticulous root excavation, manual nodule counting and weighing, and subsequent biochemical assays (e.g., acetylene reduction assay and ureide quantification) to determine fixation activity [5]. While recent developments, such as the Soybean Nodule Acquisition Pipeline (SNAP) that leverages the RetinaNet and UNet architectures, have improved quantification efficiency across diverse root architectures [11], the approaches remain destructive. This precludes the longitudinal monitoring of individual plants, introduces significant sampling variability, and renders it impractical for the large-scale phenotyping required in breeding programs or field-scale stress studies. This bottleneck underscores the urgent need for rapid, non-destructive techniques to reliably estimate key nodule parameters.

Recent advances in vegetation spectroscopy offer promising alternatives for nodule traits monitoring. Hyperspectral data capture the reflectance across contiguous narrow bands, encoding the leaf morphological and biochemical attributes through specific electronic and vibrational absorption processes [[12], [13], [14], [15]]. Critically, Ely et al. [16] demonstrated that leaf optical properties can be correlated with key traits indicative of the source-sink balance and carbon-nitrogen (C-N) status, including C and N contents, the C:N ratio, leaf mass per area, water content, and protein content, across eight diverse crop species. Extensive research further validated hyperspectral sensing for estimating critical C-N metrics such as leaf nitrogen concentration (LNC) and leaf carbon content (LCC). For instance, effective continuous wavelet transformation models were developed for monitoring soybean LCC, LNC, and C:N ratio [17]. A novel, stable method for quantifying N parameters in winter wheat was established using remote sensing data [18]. The corn leaf N status estimation was significantly improved via the spectral decomposition of high-resolution UAV imagery [19]. Recently, the feasibility of monitoring soybean LNC across multiple vertical levels and varying fertilization regimes has been confirmed [20,21]. Collectively, these studies underscore the potential of using hyperspectral sensing to infer plant traits associated with C-N physiology non-destructively, positioning it as a viable solution to overcome the limitations of destructive, labor-intensive traditional nodule assessment methods.

**Fig. 1:**
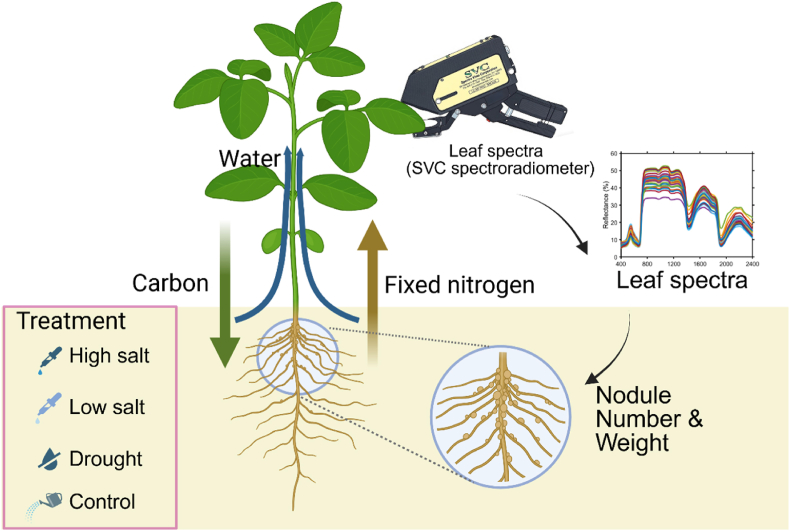
Graphic representation of the research hypothesis on the correlation between leaf spectral signatures and the underlying nodule traits (number and total weight) under abiotic stresses (salt and drought).

Building on this, we hypothesized that the profound physiological and metabolic interplay between soybean leaves and root nodules, driven by the C-N exchange and plant water status, could manifest in the distinct leaf spectral signatures that correlate with the underlying nodule traits under different abiotic stresses (Fig. 1). This hypothesis was grounded in well-established biochemical and physiological linkages. Leaves supply essential photosynthates, with approximately 9% of fixed C allocated to roots and nodules to support nodule formation and the energy-intensive N fixation process [22]. Fixed N, assimilated primarily into ureides within nodules, is translocated to the shoot [23]. This N influx can modify the leaf biochemical composition (e.g., pigment, protein, and water contents) and physical structure [24], resulting in significantly enhanced photosynthetic rates (up to 52% higher in nodulated plants) [22,25]. These functional interdependencies establish an intrinsic link between leaf physiology (e.g., photosynthesis, metabolite profiles, and hormone signaling) and root nodule status (e.g., number, biomass, and N output). Furthermore, abiotic stresses such as drought or salinity can concurrently impair both leaf functions (e.g., photosynthesis and C fixation) and nodule activities (e.g., N fixation and metabolism) by disrupting the water status throughout the plant hydraulic system [8,26,27], ultimately perturbing the symbiotic C-N exchange dynamics. Therefore, we proposed that these stress-induced perturbations in C-N exchanges would also induce quantifiable changes in leaf hyperspectral reflectance profiles. Specifically, the resultant alterations in leaf biophysical and biochemical parameters would generate detectable spectral variations across specific wavelength domains. Consequently, predictive models utilizing leaf hyperspectral data can be developed to estimate the key nodule traits (e.g., number and weight) non-destructively across diverse abiotic stress conditions.

This study aimed to address the following questions: (1) How do specific abiotic stresses (such as drought and salt) and different rhizobium strains induce characteristic alterations in soybean nodule number and weight? (2) Can leaf hyperspectral signatures provide accurate, non-destructive predictions of these nodule traits, and capture the abiotic stress-induced and rhizobium strain-attributable differences? (3) What are the underlying biological links between nodule traits and leaf reflectance spectral features? By answering these questions, we were able to establish hyperspectral leaf sensing as a novel, high-throughput methodology for the non-destructive phenotyping of soybean nodule traits and resilience under environmentally relevant stress scenarios. To our knowledge, this is the first study to establish a non-destructive, hyperspectrum-based methodology for directly quantifying soybean root nodule number and weight. This approach makes the tracking of nodule development by destructive harvesting unnecessary, and enables, for the first time, the longitudinal monitoring of nodule development in individual plants. Furthermore, by identifying the specific spectral domains and physiological linkages (e.g., water status and xanthophyll cycle) associated with nodulation under stress, we delivered novel insights into the systemic leaf-to-root communication governing symbiosis.

## 2 Materials and methods

### 2.1 Soybean materials and experimental design

To establish a robust proof-of-concept for linking leaf spectra to nodule traits, we conducted experiments in growth chambers to rigorously test the feasibility of using leaf spectral signatures for non-destructive nodule monitoring. Unlike the highly variable field settings, the controlled environment in growth chambers was essential to isolate the spectral response by precisely modulating environmental stressors and eliminating confounding field variables (e.g., weather fluctuations, heterogeneous soils, and pests). This control extended critically to the rhizobium inoculation process, eliminating the variability inherent in natural soil communities and guaranteeing uniform nodule formation, which is the prerequisite for reliably interpreting spectral signatures in this foundational study to ensure that any spectral signal detected was directly attributable to nodule development.

In this study, seeds from 18 soybean accessions were used, comprising two wild soybean lines (W05 and W06) and 16 cultivated varieties (Table S1). To better capture broad genetic and adaptive diversity, the 16 cultivated accessions were selected from those adapted to growing conditions in northern China (LH1-4, C12, ZH13, JD17, JHD1, LD7, and SP29), ones adapted to southern China (HKS-02, HKS-03), American accessions (C08 and Wm82), and Australian ones (Bragg, Bragg-*NARK* mutant). Notably, the *NARK*-mutant, which exhibits supernodulation and stunted growth due to insufficient nitrogen supply and resource trade-offs [5], would serve as a critical genetic control for studying spectral signatures. Most of the other lines exhibited moderate to strong salt tolerance, with the exception of W06, Wm82, and the *NARK*-mutant.

All seeds were surface-sterilized using chlorine gas for 16 h and germinated in sterilized vermiculite with Milli-Q water. Germination was conducted in a growth chamber under dark conditions. Four days after sowing, seedlings were transferred to a growth chamber maintained at 22-28 °C and a 16-h light/8-h dark photoperiod, supplemented with 1X B & D solution which is a nitrogen-free nutrient solution [28]. Two representative rhizobium strains, *Bradyrhizobium diazoefficients* USDA 110 and *Sinorhzobium fredii* CCBAU45436, were cultured in TY medium at 28 °C with agitation (180 rpm) for 40 h [3,29]. Prior to inoculation, the rhizobium cultures were adjusted to an optical density (OD_600_) of 0.2. Each soybean plant seedling was inoculated 4 d after sowing with 1 mL of the bacterial culture applied directly to the root zone in the vermiculite. To prevent cross-contamination, soybean lines inoculated with CCBAU45436 or USDA110 were maintained in separate growth chambers. To assess the impact of salt and drought stress on nodulation, inoculated seedlings were subjected to treatment with 500 mL of 50 mM NaCl, 150 mM NaCl or 15% PEG-6000, respectively, administered three times per week. There were four biological replicates for each soybean line under each treatment condition. At 21 days post-inoculation (dpi), the soybean plants were harvested for data collection.

### 2.2 Data collection

#### 2.2.1 Leaf spectra collection

Leaf spectral measurements were taken uniformly from the central leaflet of the first fully expanded trifoliate leaf on every plant. Leaf reflectance was measured using a field-portable HR-1024i spectroradiometer (Spectra Vista Corporation [SVC], Poughkeepsie, NY, USA) equipped with a Leaf-Clip Reflectance-Probe (LC-RP-Pro) fore-optic. Spectral measurements were taken from 350 nm to 2500 nm. The instrument had a full-width half maximum (FWHM) of approximately 3.3 nm at 700 nm, 9.5 nm at 1500 nm, and 6.5 nm at 2100 nm. An internal tungsten halogen lamp within the probe illuminated the leaf sample against a dark background. Reflectance was calibrated against a 99% reflective Spectralon white reference panel (Labsphere Inc., North Sutton, NH, USA) prior to each measuring session. The spectrometer was configured with a 1-s integration time to minimize potential heat-induced data degradation [30]. Spectral regions below 400 nm and above 2400 nm, characterized by higher noise levels, were excluded from subsequent analyses [15]. Vendor-provided software was used to correct the raw spectra for discontinuities [12]. For each leaf, reflectance data were acquired from three distinct locations on the adaxial surface, with three replicate scans recorded at each location. The resulting nine spectra per leaf were then averaged to yield a single representative spectral signature for analysis.

#### 2.2.2 Nodule traits measurement

Immediately following spectral measurement, the entire root system of each plant was carefully excavated from the vermiculite growth medium. Roots were gently rinsed with Milli-Q water to remove any adhering substrate. The root system was then placed on clean, dry paper towels and rolled gently for 3–5 s to remove excess surface moisture without compressing the nodules. Individual nodules were manually excised, placed on a fresh, dry area of the paper towel, and given a final brief blot (< 2 s). Total nodule fresh weight per plant was determined using an analytical balance (ATX24, 220 g: 0.1 mg; Ruich Allway [China] Ltd.). Nodule fresh weight was used as a practical, non-destructive metric that reflects functional turgor and metabolic status [31]. The entire process from the removal from growth medium to the final weighing was completed within 1 min under ambient laboratory conditions (∼26 °C) to minimize tissue water loss.

### 2.3 Data analysis

Aiming to investigate the relationship between leaf spectra and nodule traits (number and weight) under different rhizobium strains and abiotic stresses, we first quantified the impacts of drought and salt stress as well as rhizobium strains USDA110 and CCBAU45436 on nodule numbers and total weights across diverse soybean genotypes. Next, we investigated the associations between these traits and leaf spectra and developed predictive models, to evaluate the feasibility of using leaf hyperspectral data for the non-destructive estimation of nodule number and weight under stress. Finally, we explored the underlying biological connections between leaf spectra and nodule traits.

#### 2.3.1 Quantifying stress- and strain-induced nodule trait variations

Statistical analyses assessing the effects of abiotic stresses (salt, drought) and rhizobium strains on soybean nodule number and weight were conducted using GraphPad Prism 5 (GraphPad Software Inc., San Diego, CA, USA). Data were analyzed by one-way ANOVA followed by Tukey's *post hoc* test.

#### 2.3.2 Exploring the associations between nodule traits and leaf spectra

To investigate the relationship between leaf spectra and nodule traits (number and weight), we employed a rigorous analytical pipeline incorporating spectral preprocessing and Partial Least Squares Regression (PLSR) modeling. Raw leaf spectra were subjected to five common preprocessing algorithms to mitigate noise and enhance signal quality prior to modeling, including Savitzky-Golay smoothing (SG; with window size of 11 and polynomial order of 2), standard normal variate (SNV), Z-Score standardization (ZS), first-order derivative (FD) and second-order derivative (SD) [[32], [33], [34], [35], [36]]. PLSR was then applied to model the quantitative relationship between preprocessed leaf spectra and nodule traits (number and weight) across various rhizobium strains and drought and salinity stress, using our comprehensive spectral-phenotypic database (*n* = 528). PLSR is particularly suited for spectroscopic data analysis as it performs dimensionality reduction akin to Principal Component Analysis (PCA), and can maximize the covariance between predictor (spectra) and response (nodule trait) variables, while maintaining the interpretability of linear regression [12,[37], [38], [39]]. To ensure robust model performance and minimize overfitting, we implemented a stratified repeated double cross-validation (rdCV) PLSR modeling approach [40]. Briefly, for the outer loop, the full dataset was randomly partitioned into calibration (90%) and independent validation (10%) subsets for 100 times using 10-fold cross-validation. The stratification was applied at this stage to ensure each of the 10 folds had a similar distribution of the key grouping variables (genotype × treatment). This ensured that each subset (both calibration and validation) maintained a proportional representation of the different biological and experimental conditions present in the full dataset. This strategy was crucial to prevent a scenario where, for example, all plants of a specific genotype or under a specific stress treatment were grouped into only the calibration or only the validation set, which would lead to biased performance estimates.

For each outer loop iteration, the calibration subset was further split (10-fold CV) into training and testing sets. This inner loop determined the optimal number of latent variables (LVs) by maximizing the average coefficient of determination (R^2^) and minimizing the average Root Mean Square Error (RMSE) on the testing sets. Within the inner loop, Variable Importance in Projection (VIP) scores were calculated for the model using optimal LVs. Wavelengths with VIP >1 were considered significantly influential [41]. A final average VIP spectrum was generated across all inner loop iterations. Using the optimal number of LVs identified in the inner loop, a final PLSR model was built on the entire calibration subset and evaluated on the held-out validation subset within each outer loop iteration. The use of stratified, repeated double cross-validation is a robust approach designed precisely to mitigate overfitting and provide a realistic estimate of model performance on new, unseen data that may come from any of the represented genotypes or treatments.

Model performance was assessed using the R^2^ for linear regressions between observed and predicted values, and the RMSE and normalized RMSE (nRMSE) of prediction within independent validation subsets. This process yielded an ensemble of 100 independent PLSR models for each nodule trait. Next, we reconstructed PLSR models for each of the two rhizobium strains (USDA110 and CCBAU45436) and compared their diagnostic spectral domains (VIP >1) and cross-validation results to assess generalizability. Concurrently, the consistency and specificity of the PLSR-derived diagnostic domains under drought and salt stress were assessed by performing PLSR modeling for each stress condition separately, which can then be used to pinpoint the key wavelengths capturing unique physiological adaptations and validate leaf spectra as reliable proxies for abiotic stress responses. As the final step, we demonstrated the applicability of using the established spectra-derived PLSR models for the real-time monitoring of nodule traits.

#### 2.3.3 Investigating the underlying physiological links between nodule traits and leaf spectra

The symbiotic C-N exchange between root nodules and leaves fundamentally alters the leaf biochemistry (e.g., chlorophyll, protein and water contents) and structure (e.g., mesophyll cell morphology), directly modulating leaf optical properties. To establish a mechanistic understanding of the linkages between leaf spectra and nodulation, a dual-proxy analytical framework was employed to integrate both empirical vegetation indices (VIs) and specific leaf biophysical traits through radiative transfer modeling (RTM) inversion. We computed 19 established VIs (Table S2) targeting chlorophyll (proxy for N status), carotenoids/xanthophylls (photoprotection), water content, and photosynthetic efficiency to capture biochemical shifts altered by C-N dynamics. Concurrently, key leaf biophysical traits, including chlorophyll *a*+*b* content (Cab), carotenoid contents (Car), anthocyanin content (Anth), brown pigment contents (Cbrown), and equivalent water thickness (EWT) were quantified, by inverting the full-range (400-2500 nm) leaf spectral data. This inversion was performed using the PROSPECT-5 RTM [[42], [43], [44], [45]], which simulates leaf reflectance and transmittance based on its internal light-scattering properties and the complex refractive indices of its constituent biochemicals. The inversion process was implemented in MATLAB R2024a using a trust-region reflective optimization algorithm to iteratively minimize the residuals between the observed reflectance spectra and those modeled by PROSPECT-5 (Fig. S1), thereby deriving the most probable trait values for each sample. This algorithm enables the inversion of the PROSPECT + COSINE model through simulated bidirectional reflectance factor measurement and iterative optimization, allowing us to estimate these biophysical traits. Subsequently, Pearson correlation analysis was applied to evaluate the relationships between the two nodule traits (number and weight), and both sets of spectral proxies: the empirical VIs and the mechanistically retrieved biophysical traits. This analysis was conducted across different rhizobium strains and abiotic stress treatments. By integrating these two complementary approaches, our framework effectively discriminated against dominant physiological processes, such as pigment dynamics, water status, and structural changes, that govern the observed spectral linkages to root nodule development.

## 3 Results

### 3.1 Nodule traits varied depending on rhizobium strains and abiotic stress types

To assess the feasibility of utilizing leaf spectral signatures to monitor soybean nodule traits non-destructively, we employed a diverse set of soybean genotypes with varying tolerance levels to salt and drought stress, ensuring broad biological relevance for the subsequent models to be built based on the data obtained from these treatments. Comprehensive statistics for the average nodule numbers and total nodule weights across all genotypes and treatments are detailed in Tables S3 and S4, respectively.

**Fig. 2:**
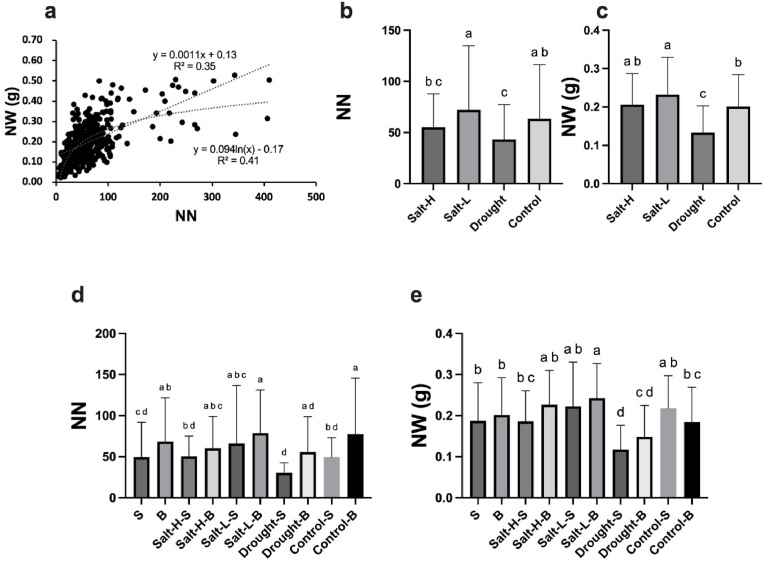
Nodule number was positively correlated to total nodule weight and was affected by the abiotic stress type and rhizobium strain. (a) The linear and logarithmic correlations between nodule number (NN) and total nodule weight (NW). R^2^, coefficient of determination in linear/logarithmic regression. (b-e) Nodule number (b and d) and total nodule weight (c and e) of soybean lines across salt and drought stresses. (b-c) Nodule number (b) and total nodule weight (c) of soybean lines inoculated with rhizobium under salt and drought stresses. Salt-H, high-concentration salt treatment (150 mM); Salt-L, low-concentration salt treatment (50 mM); Drought, PEG-6000 treatment; S, rhizobium strain CCBAU45436-inoculated; B, rhizobium strain USDA110-inoculated. One-way ANOVA was performed, followed by a *post hoc* Tukey test. Different letters above the bars indicate the corresponding means are significantly different (*P* < 0.05).

Analyses of the nodulation phenotype across treatments revealed a significant positive relationship between nodule number and total nodule weight. Both linear regression (R^2^ = 0.35, p < 0.001) and logarithmic regression (R^2^ = 0.41, p < 0.001) models indicated that higher nodule numbers generally corresponded to greater total nodule biomass (Fig. 2a). Nodulation was also profoundly impacted by the abiotic stress type and severity. PEG-6000-induced drought stress caused the most severe overall suppression of nodulation, resulting in the lowest combination of nodule numbers and total nodule weights (Fig. 2, b-c). High-concentration salt stress (150 mM NaCl) significantly reduced nodule numbers compared to low-concentration salt treatment, similar to the effect of drought treatment (Fig. 2b). Notably, total nodule weight under low-concentration salt stress (50 mM NaCl) was higher than both the non-stressed control and drought stress (Fig. 2c). Crucially, while high-salt stress significantly reduced nodule numbers compared to low-salt stress, no significant difference in the total nodule weight was observed between these two salt treatments (Fig. 2c). This indicates that low-concentration salt stress disrupted the nodulation process minimally in most of the soybean lines tested, potentially reflecting inherent salt tolerance.

Significant differences in nodulation efficiency were observed between inoculating with the two rhizobium strains under certain abiotic stress conditions (Fig. 2, d-e). Plants inoculated with USDA110 consistently developed significantly more nodules than those inoculated with CCBAU45436 under drought treatment and in unstressed controls, and had a slight, although not statistically significant, advantage under both high- and low-salt conditions (Fig. 2d), highlighting USDA110's superior nodulation capacity. Drought stress tended to exert the strongest suppressive effect on both nodule number and total nodule weight, regardless of the inoculating strain (Fig. 2d–e). Importantly, however, the USDA110-associated increase in nodule number did not lead to a corresponding proportional increase in total nodule weight compared to inoculating with CCBAU45436 (Fig. 2e). This demonstrates that rhizobium strain variation primarily modulated the nodule number, with a less pronounced direct effect on the total weight of nodules per plant within this dataset. Overall, the divergent nodulation responses elicited by varying stress types and intensities (drought > high salt > low salt/control) and the distinct phenotypes conferred by the two different rhizobium strains collectively provided a robust phenotypic gradient. This wide range of nodule trait variations under controlled experimental conditions thus formed a powerful biological basis for developing and training spectral prediction models.

### 3.2 Spectral prediction models of nodule traits

**Fig. 3:**
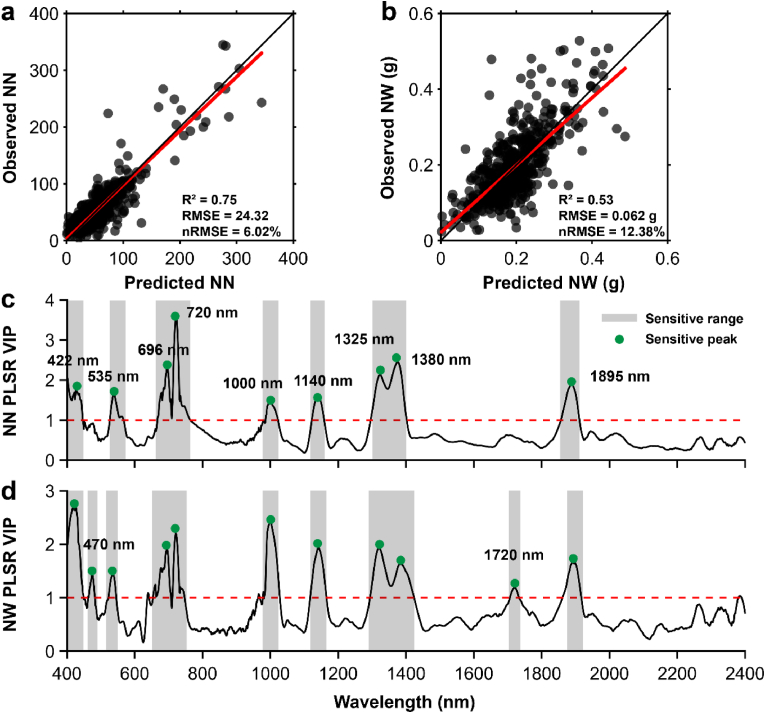
Partial Least Squares Regression (PLSR) model performance and the variable importance projection (VIP) of the PLSR model. (a) Model performance with respect to nodule number (NN) prediction; (b) model performance with respect to total nodule weight (NW) prediction; (c) PLSR VIP of NN; and (d) PLSR VIP of NW. R^2^, coefficient of determination; RMSE, root mean square error; nRMSE, normalized mean square error. Red lines in (a) and (b) represent the actual regression results whereas black diagonal lines represent a theoretical 1:1 correlation between the predicted and observed values of the nodule traits. Red dotted lines in (c) and (d) indicate a VIP value of 1. The spectral regions above the dotted lines were identified as significant.

To address the associations between nodule traits and leaf spectra, we conducted PLSR modeling analyses. Preprocessing optimization revealed that Savitzky-Golay smoothing combined with Z-Score standardization and first-derivative transformation (SG-ZS-FD) yielded superior performance over raw spectral data (Table 1) for both nodule number and total nodule weight predictions. Specifically, the leaf spectra-nodule number PLSR model demonstrated a strong predictive power, with an R^2^ of 0.75, an RMSE of 24.32 and an nRMSE of 6.02% (*n* = 528, Fig. 3a). The leaf spectra-nodule weight PLSR model could also infer nodule weight with good accuracy (*n* = 528, R^2^ = 0.53, RMSE = 0.062 g, nRMSE = 12.38%, Fig. 3b). The significant difference in predictive performance between the two spectral models (notably the lower R^2^ for weight) was primarily attributable to the comparatively reduced variability (narrower data distribution range) observed within the nodule weight dataset (Fig. 2a). To further explore the sensitive hyperspectral signatures of nodule number and weight, we deployed Variable Importance in Projection (VIP) scores to identify their associated significant spectral regions (indicated by VIP >1). Notably, both the leaf spectra-nodule number PLSR model and the leaf spectra-nodule weight PLSR model revealed similar significant spectral regions with nine pronounced spectral peaks around 422 nm, 535 nm, 696 nm, 720 nm, 1000 nm, 1140 nm, 1325 nm, 1380 nm and 1895 nm, respectively (Fig. 3, c-d), stemming from their co-linearity (Fig. 2a). However, in addition to these common peaks, the leaf spectra-nodule weight PLSR model presented two unique sensitive spectral domains, featuring peaks at 470 nm and 1720 nm, respectively (Fig. 3d).

**Table 1:** Partial Least Squares Regression (PLSR) model performance for the estimation of nodule number and nodule weight with different preprocessing optimization methods (*n* = 528).

	Nodule number			Nodule weight		
	R^2^	RMSE	nRMSE	R^2^	RMSE	nRMSE
Raw	0.43	36.89	9.13%	0.25	0.078	15.48%
SG	0.61	30.39	7.52%	0.35	0.074	14.68%
SG + SNV	0.61	30.69	7.59%	0.34	0.074	14.68%
SG + ZS	0.63	29.73	7.36%	0.37	0.072	14.29%
SG + ZS + FD	**0.75**	**24.32**	**6.02%**	**0.53**	**0.062**	**12.38%**
SG + ZS + SD	0.33	40.02	9.91%	0.26	0.078	15.48%

RMSE, Root Mean Square Error; nRMSE, normalized RMSE; SG, Savitzky-Golay smoothing; SNV, standard normal variate; ZS, Z-Score standardization; FD, first-order derivative; SD, second-order derivative.

**Fig. 4:**
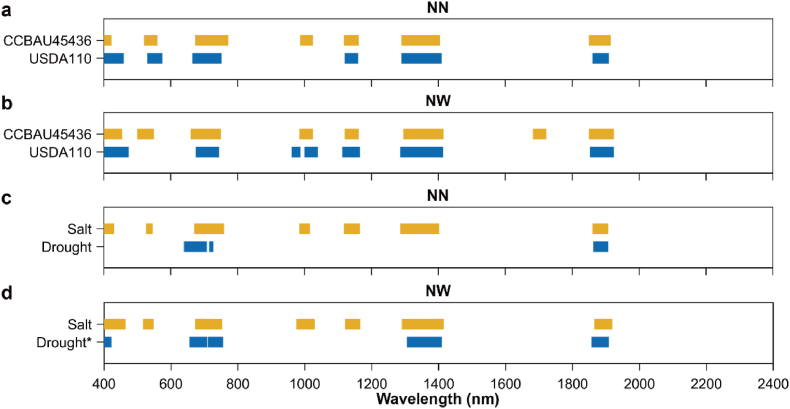
Optimal spectral domains generated by the Partial Least Squares Regression (PLSR) model for (a) nodule number (NN) and (b) total nodule weight (NW) across distinct rhizobium strains (CCBAU45436 or USDA110) and for (c) NN and (d) NW across abiotic stress (salt or drought). ∗ indicates the coefficient of determination (R^2^) of the leaf spectra-NW PLSR model for drought in (d) was lower than 0.25.

Next, to evaluate the strain-specificity of our spectral models, we reconstructed a separate PLSR model for each rhizobium strain (USDA110 and CCBAU45436), and the performance metrics for these models are detailed in Table S5. Notably, the optimal diagnostic spectral domains identified for predicting both nodule number and weight were highly consistent between the two strains. Both models highlighted the importance of shared spectral regions at 400-420 nm, 675-745 nm, 1120–1160 nm, 1295–1405 nm, and 1860–1910 nm, respectively (Fig. 4a–b, Table S6). This convergence in informative wavelengths suggested that the physiological mechanisms linking leaf spectra to nodulation may be conserved across these strains, indicating that a unified PLSR model could be robust enough for trait prediction irrespective of the rhizobium partner. We then tested this hypothesis of generalizability directly through a cross-validation scheme. The predictive power for nodule number was maintained when applying the CCBAU45436-derived PLSR model to the USDA110-inoculated dataset (R^2^ = 0.31, RMSE = 50.60) and vice versa (R^2^ = 0.31, RMSE = 43.45) (Fig. S2, a and c). This reciprocal validation confirms the model's applicability across strains for this trait. In contrast, the predictive accuracy for total nodule weight was poor in both cross-validation scenarios (Fig. S2, b and d), indicating that the spectral signatures associated with this trait may be more sensitive to strain-specific influences. For example, we identified two distinct spectral regions at 500-550 nm and 1683–1723 nm for predicting total nodule weight with CCBAU45436 (Fig. 4b).

Furthermore, to identify stress-specific diagnostic wavelengths, we evaluated the consistency and specificity of PLSR-derived influential spectral domains under salt and drought stress. Both stresses exhibited significant spectral responses in the near-infrared (NIR) region, with salt stress uniquely characterized by four key domains at 520-545 nm, 984-1030 nm, 1120–1166 nm, and 1290–1415 nm, respectively (Fig. 4c). These signatures reflected distinct physiological adaptation mechanisms between salt and drought stress, thereafter altering leaf optical properties and manifesting as stress-specific spectral fingerprints. Overall, these results demonstrated strong associations between leaf spectra and nodule traits, confirming that leaf spectra alone can be used to quantify nodule traits non-destructively.

**Fig. 5:**
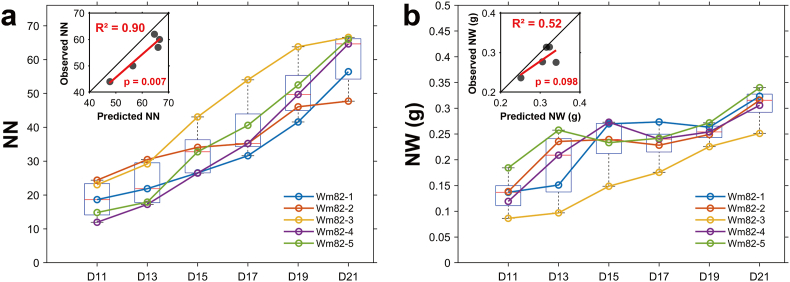
Demonstration of real-time and *in-situ* monitoring of (a) nodule number (NN) and (b) total nodule weight (NW) for individual soybean plants after inoculation with the rhizobium strain CCBAU45436 by integrating leaf spectral data with Partial Least Squares Regression (PLSR) models. Wm82-x indicates the genotype (Wm82) and sample number (x). X-axis indicates the number of days post-inoculation.

Finally, to validate the suitability of leaf spectra for time-series (i.e. continuous) and *in-situ* monitoring of nodule development, we deployed a real-time, non-destructive spectral phenotyping pipeline. This system leveraged our established PLSR models to track nodule trait dynamics in individual plants over time. For this validation, we selected five plants of the Wm82 genotype, a model system whose trait values (nodule number: 0-100; weight: 0-0.4 g) fall entirely within the calibration range of our models, and cultivated them under optimal conditions. Following inoculation with the CCBAU45436 strain when the soybean plants were 10 days old, we acquired leaf spectral data every 2 d until 21 dpi, when the plants were harvested for final nodule trait validation. Our approach successfully captured the temporal progression of both nodule number and weight (Fig. 5) with high sustained accuracy for nodule number (R^2^ = 0.90, *p* = 0.007) and moderate accuracy for total nodule weight (R^2^ = 0.52, *p* = 0.098), demonstrating the potential for continuous, non-destructive surveillance of nodulation status. Crucially, this longitudinal design revealed divergent growth strategies among genetically identical plants. For example, plant Wm82-3 exhibited the highest nodule number but the lowest total nodule weight and a sustained increase in both traits throughout the monitored period. In contrast, the other four plants displayed a continued increase in nodule numbers after 15-19 dpi but reached a plateau in nodule weight during the same period, suggesting a potential shift in resource allocation. Further research on the exploration of the synergetic growth dynamics of nodule traits is still needed. Overall, this compelling demonstration underscored the transformative potential of hyperspectral phenotyping as a powerful tool for functional genomics. It provided a critical complementary methodology to further explore the genetic and environmental drivers of nodulation strategies, opening new avenues to investigate the complex biological connections between plant genotypes, rhizobial symbionts, and the resulting phenotypic plasticity in nodulation status.

### 3.3 The potential links between nodule traits and leaf spectra

Our PLSR models revealed that sensitive spectral domains for nodule number and total nodule weight overlapped with key domains governing C-N exchange dynamics, notably chlorophyll degradation (680-720 nm), xanthophyll cycle activity (520-545 nm), and leaf water status (980-1150 nm, 1290–1405 nm, and 1850–1950 nm). This spectral congruence suggested that nodule development alters leaf optical properties through biochemical pathways analogous to the C-N-related processes. Therefore, to dissect these linkages, we quantified the relationships between nodule number/weight and 19 VIs, targeting chlorophyll, carotenoids, and photosynthetic efficiency, alongside five leaf biophysical traits inferred by PROSPECT-5, including chlorophyll *a*+*b* content (Cab), carotenoids content (Car), anthocyanin content (Anth), brown pigments content (Cbrown), and equivalent water thickness (EWT) across rhizobium strains (USDA110 and CCBAU45436) and abiotic stresses (salt and drought).

**Fig. 6:**
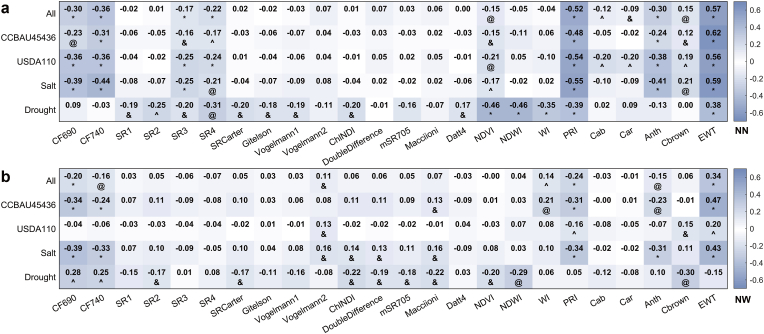
Pearson correlation coefficients (*r*) between 19 empirical vegetation indices (VIs), five PROSPECT-derived leaf biophysical traits and two nodule traits: (a) nodule number (NN) and (b) total nodule weight (NW). Correlations (*r* values) are shown for the full dataset (All) and are further differentiated across key experimental conditions: rhizobium strains (CCBAU45436 and USDA110) and abiotic stress (drought and salt). Symbols below the *r* values indicate levels of statistical significance as determined by the Student's t-test (&, *p* < 0.05; ^, *p* < 0.01; @, *p* < 0.001; ∗, *p* < 0.0001). Detailed descriptions of the VIs are listed in Table S2. Cab, chlorophyll *a*+b content; Car, carotenoids content; Anth, anthocyanin content; Cbrown, brown pigments content; EWT, equivalent water thickness. The color scale to the right of each panel is proportional to the absolute value of the correlation coefficient (|*r*|).

Nodule traits exhibited significant spectral linkages (*p* < 0.0001) characterized by a positive correlation with EWT and negative correlations with other key VIs, including chlorophyll fluorescence indices (CF690 and CF740), and photochemical reflectance index (PRI), reflecting xanthophyll cycle modulations (Fig. 6). Notably, nodule numbers showed generally stronger associations (|*r*| = 0.30 − 0.62) than nodule weight (|*r*| = 0.20 − 0.47) with these VIs or EWT, indicating greater spectral sensitivity to nodule initiation than biomass accumulation. Specifically, the positive EWT correlation (*r* = 0.34 − 0.62) implied that nodule development can enhance leaf water retention, while negative VI correlations (CF690: *r* = −0.39 − −0.20; CF740: *r* = −0.44 − −0.24; PRI: *r* = −0.55 − −0.2) indicated that nodule development was highly correlated to C-N reallocation in the leaf under the various test conditions or with different rhizobium strains (Fig. 6). In addition, both rhizobium strains exhibited comparable correlations between VIs/EWT and nodule number (e.g., PRI: *r* = −0.54 for USDA110 vs. *r* = −0.48 for CCBAU45436, Fig. 6a), while for total nodule weight, USDA110 showed no significant spectral links to most of the VIs/EWT (Fig. 6b), suggesting rhizobium strain-specific differences in C-N reallocation during biomass accumulation. In particular, the correlations with PRI and EWT were significantly stronger under salt stress than those under drought stress for both nodule traits, and correlations with chlorophyll fluorescence indices (CF690 and CF740) were also significantly stronger for nodule number under salt stress than under drought. Overall, the robustness of CF690, CF740, PRI and EWT as predictors of nodule traits (*p* < 0.0001 across rhizobium strains and abiotic stresses) confirmed their utility for non-destructive monitoring of the structural determinants of symbiotic potential, while rhizobium strain- and stress-dependent variations further underscored the need for context-specific spectral models.

## 4 Discussion

This study deciphered the abiotic stress- and rhizobium strain-induced characteristic alterations in soybean nodule traits and their impacts on leaf spectra. Our results revealed significant variability in nodule traits across two representative rhizobium strains and drought and salt stress. Specifically, drought showed a stronger effect on nodulation than salt stress (Fig. 2, b-c). Furthermore, our study demonstrated that leaf hyperspectral reflectance enabled the non-destructive quantification of nodule number and total nodule weight, with the spectral domains identified by PLSR models demonstrated to be robust across rhizobium strains (Fig. 4a–b). Stress-specific spectral fingerprints uniquely captured distinct physiological adaptations via altered leaf optical properties under salinity and drought (Fig. 4, c-d). Further investigations into spectral linkages revealed nodule-driven shifts in leaf water retention and leaf C-N exchanges,by identifying the underlying associations between nodule traits and chlorophyll, xanthophyll and EWT, explaining the differences in leaf spectral signatures between the two nodule traits under different treatment conditions and with different rhizobial inoculants (Fig. 6). All these observations, when taken together, provided a comprehensive investigation of the associations between leaf spectra and nodule traits across different rhizobium strains and abiotic stresses, highlighting the fundamental biological linkages underlying this integrative spectral-physiological framework for the non-destructive phenotyping of nodule traits.

### 4.1 Stress induced the variations in nodule traits

Our experimental results demonstrated that both salt and drought stress exerted significant inhibitory effects on soybean nodulation, as evidenced by reductions in nodule number and weight (Fig. 2, b-c). Notably, the impact of rhizobium strain selection emerged as a critical factor, with USDA110 exhibiting superior nodulation capacity compared to CCBAU45436 (Fig. 2d). This observation aligned with prior findings [46], reinforcing the strain-dependent nature of symbiotic efficiency under abiotic stress conditions. PEG-6000-induced drought stress caused more severe nodulation impairment than salt stress (Fig. 2, b-c), suggesting that water deficiency may pose a greater threat to nodulation status in soybean than moderate salinity. Low-concentration salt stress (50 mM NaCl) did not significantly reduce nodulation, likely a result of the combined effects of (1) the inherent salt tolerance of the soybean genotypes used in this study and (2) the alkaline-saline adaptations by CCBAU45436, which partially mitigated the salt-induced suppression of nodule formation (Fig. 2d). These findings carried substantial practical implications for sustainable soybean cultivation in marginal environments. In semi-arid or saline regions, the selection of salt-tolerant soybean lines alone may be insufficient to ensure optimal nodulation. Our data highlighted that water availability remains the primary limiting factor, even in salinity-adapted systems. The strain-specific responses underscored the need for tailored rhizobium inoculants, with USDA110 representing a promising candidate for drought-prone areas.

### 4.2 Mechanisms linking spectral data to nodule traits

Given the unique sensitivity of nodule traits to various abiotic stresses and rhizobium strains, as well as the practical importance of non-destructive phenotyping, we have demonstrated that leaf hyperspectral sensing is a robust and non-invasive method for quantifying soybean nodule traits. Notably, we identified novel diagnostic wavelengths directly associated with nodule development. For example, both nodule number and total nodule weight showed strong and consistent spectral associations across several regions, including three peaks in the visible range (422 nm, 535 nm and 696 nm) and additional peaks in the near-infrared and shortwave-infrared ranges (720 nm, 1000 nm, 1140 nm, 1325 nm, 1380 nm and 1895 nm) (Fig. 3, c-d). Crucially, these spectral domains, previously unreported due to the scarcity of paired spectral-nodule datasets, reflected fundamental biophysical processes such as chemical bond vibrations (e.g., C-H, N-H and O-H bonds; [47,48]), photosynthetic reallocation involving chlorophyll (450-650 nm and 687 nm) and xanthophyll (400-531 nm), key biochemical traits associated with C-N exchanges, including LCC, LNC, N_area_ and N_mass_ at wavelengths such as 649 nm, 650 nm, 678-728 nm, 800-1100 nm and 1700–1900 nm, and hydraulic dynamics such as leaf water content (near 1450 nm and 1950–1980 nm) [13,17,[49], [50], [51], [52], [53]]. These findings underscored that profound physiological and metabolic interplay between soybean leaves and root nodules, primarily driven by C-N exchanges and water cycling, and manifesting as distinct leaf spectral signatures that correlate with key nodule traits. However, a further exploration to decipher the direct association between spectral signatures and leaf C, N, and H should be conducted via direct isotopic analysis (e.g., δ^13^C, δ^15^N, δ^2^H).

In this study, a striking similarity was observed in optimal spectral domains across rhizobium strains, while cross-validation indicated that the nodule number model exhibited some transferability between different strains (R^2^ = 0.31), whereas the nodule weight model showed poor transferability. These contrasting degrees of transferability between the nodule number and nodule weight models across rhizobial strains likely stem from their distinct physiological determinants. Nodule number is primarily regulated by conserved host genetic pathways involved in early symbiosis and autoregulation of nodulation (AON), making it relatively resistant to variations in rhizobial identity [54,66]. Once nodules are initiated, the onset of nitrogen fixation triggers systemic changes in leaf nitrogen and carbon statuses, generating spectral signals detectable by our PLSR model with moderate generalizability across strains. In contrast, total nodule fresh weight integrates strain-specific symbiotic performance, a reflection of the cumulative outcome of carbon allocation to nodules, which in turn is tightly coupled to the efficiency of the carbon-for-nitrogen exchange between the host and the microsymbiont. Different rhizobium strains establish unique physiological equilibria with the host, modulating photosynthate partitioning and the associated leaf metabolic profiles [55]. Consequently, the spectral signatures linked to nodule biomass are more intimately tied to the specific plant–rhizobial partnership, limiting direct model transferability. This underscores that while nodule number can be predicted from generalized stress or symbiosis responses, the accurate, non-destructive estimation of nodule biomass may require a calibration for specific rhizobial partnerships or necessitate the use of models that incorporate proxies for symbiotic efficiency.

On the other hand, our study revealed distinct hyperspectral signatures under salt and drought stress (Fig. 4). Although direct physiological measurements were not conducted, the observed spectral patterns aligned with well-documented, stress-specific mechanisms. Under salt stress, plants face several physiological challenges, including osmotic stress, ion toxicity, and oxidative damage. Ion toxicity (Na^+^/Cl^−^ accumulation) disrupts ion homeostasis, causing nutrient imbalance and metabolic dysfunction [56]. Hence, this may lend greater importance to the spectral bands related to ion toxicity (e.g., Na^+^ and Cl^−^ effects) under salt stress. For example, Ignat et al. [57] reported that the most responsive wavelengths for Cl^−^ were 500-531 nm, 682-752 nm, 1310–1407 nm, and 1768 nm, and those for Na^+^ were 500-531 nm and 682-752 nm. These ranges aligned closely with the unique spectral domains we identified for salt stress (520-545 nm, 984-1030 nm, 1120–1166 nm, and 1290–1415 nm; Fig. 4, c-d). This indicated that analyzing leaf spectral differences can reveal the pronounced effects of ion toxicity under salt stress. In contrast, drought-sensitive signals were concentrated in two key regions (640-728 nm and 1863–1908 nm), which primarily correspond to red light and red edge, as well as water-related spectral bands. These are notably similar to the wavelength sensitive to stomatal conductance, such as 716 nm and 1854 nm, which were identified by Cheng et al. [12]. This suggested that the primary physiological mechanism driving the spectral response to drought was related to stomatal regulation, consistent with established plant physiological principles [[58], [59], [60]]. Our findings demonstrated that the spectral divergence directly captures stress-specific physiological adaptations, adding to our confidence that leaf spectra can provide quantifiable signatures of these distinct mechanisms.

In the meantime, we agree that distinguishing stress response signals from nodulation signals is paramount. However, our finding that the diagnostic spectral domains for nodule number were remarkably consistent between the two rhizobium strains (Fig. 4a–b) suggests that these features are tied to the nodulation process itself. If the model were primarily learning the stress status, we would expect more divergence between strains, as they can induce different physiological states. Hence this convergence between the rhizobium strain-specific models points to a common nodule-driven spectral signature. Furthermore, the PLSR model identified unique spectral regions specific to nodule weight that were not key for predicting nodule number (Fig. 3, c-d). This trait specificity also indicates that the models are resolving fine-scale physiological differences beyond a broad stress response.

Following these observations, we next assessed the associations between nodule traits and spectra-derived VIs and EWT across rhizobium strains and abiotic stresses quantitatively to further dissect their underlying linkages. We discovered positive correlations between the water-related index, EWT, and nodule traits, and negative associations between pigment-related VIs (e.g., CF690 and PRI) and nodule traits (Fig. 6). One possible reason for the positive nodule trait-EWT correlations could have stemmed from osmotic adjustments during N assimilation [31]. Nodule activities produce N-rich compounds (e.g., ureides), which are then transported to the shoot, thus lowering the cellular osmotic potential and enhancing the water retention capacity in leaves, thereby elevating EWT. Besides, we also observed generally stronger associations of nodule traits to salt stress than to drought (Fig. 6), likely ascribable to nodule development being more sensitive to the osmotic stress component of elevated salinity [31]. Furthermore, the observed negative correlations between nodule traits and photosynthetic pigment-related VIs (e.g., CF690 and PRI) can be attributed to nutrient limitations, particularly that of N. Our experiments were designed to intentionally limit N supply in order to stimulate nodule formation. This resulted in the competitive allocation of N and C resources between leaves and nodules, as evidenced by the negative correlations between pigment-related VIs and nodule traits. Collectively, this finding suggested that the ability of leaf spectra to reflect nodule development under stress may be linked to the abiotic stress-induced changes in internal leaf properties (such as chlorophyll [CF690], xanthophyll [PRI] and leaf water content [EWT]) that have resulted from nodule activities that were modulated in response to abiotic stress.

### 4.3 Analysis of model performance variations

Beyond elucidating the potential mechanisms linking spectral features and nodule traits, our results established single spectral models as a high-throughput, non-destructive solution for nodule phenotyping, achieving robust predictive accuracy (R^2^ = 0.75 for nodule number, R^2^ = 0.53 for total nodule weight, Fig. 3, a-b). The superior predictive performance of the model for nodule number compared to that for fresh nodule weight was likely due to the fundamental nature of these traits. Nodule number is a stable, structural metric that integrates the developmental history of the symbiosis. In contrast, fresh nodule weight is a dynamic physiological trait, highly sensitive to transient changes in plant water status and environmental conditions, such as soil moisture and plant health status, and the spatial distribution of nodules on the roots and measurement procedures. The higher susceptibility of nodule weight to these sources of variability introduces greater noise into the dataset, which is reflected in the relatively moderate predictive accuracy of the model. However, this does not diminish the utility of the weight prediction but accurately reflects the biological and methodological realities of measuring this particular trait. To further improve model precision, future research could explore fusing spectral data with other sensor modalities (e.g., thermal or fluorescence imaging) or leverage advanced machine/deep learning techniques that can account for these complex, non-linear interactions. Nonetheless, this approach notably overcame the longstanding challenge of *in-situ* nodule monitoring, as validated by the longitudinal tracking of individual plants (Fig. 5). Our models underscored hyperspectral sensing as a transformative tool for the real-time quantification of nodule development dynamics, with profound implications for sustainable agriculture and crop research. By enabling non-destructive screening, this high-throughput method can accelerate the selection of stress-resilient soybean-rhizobium pairs without destructive harvests, potentially reducing the number of breeding cycles required [61]. In conjunction with our findings, hyperspectral sensing can not only help select for abiotic stress resilience, but it can also be potentially applied to the detection of biotic stressors such as pathogens and pests [62,63]. Furthermore, it can facilitate the matching of superior soybean genotypes with elite rhizobium strains. This dual capability helps drive sustainable agriculture. Farmers can optimize nitrogen fixation and reduce their reliance on chemical fertilizers, thus lowering production costs while increasing yields.

### 4.4 Field application potential and challenges of leaf hyperspectral sensing

While leaf-level hyperspectral sensing shows strong potential for the non-destructive monitoring of nodule traits, translating this approach from controlled growth chambers to field conditions introduces significant additional complexity. Key challenges include scaling up from leaf to canopy, accounting for soil heterogeneity, and managing climatic variability, all of which can affect model stability. Inside growth chambers, spectral measurements are taken from individual leaves under consistent illumination, minimizing variability. In the field, canopy-level spectra integrate signals from multiple leaves, shadows, soil background, and plant structure, which can obscure the spectral signatures linked to nodule traits. Signals at the sensitive wavelengths identified in our study—such as the 720 nm peak associated with chlorophyll degradation or the 1895 nm peak related to water content—may be diluted due to mixed pixels and bidirectional reflectance effects. To address this, future work should employ radiative transfer models (RTMs) such as PROSAIL [64], which simulate canopy reflectance by integrating leaf-level properties (e.g., from PROSPECT-5 inversion) and canopy structure parameters. Insights from our leaf-level study, such as the correlation between nodule number and equivalent water thickness (EWT), can serve as the prior knowledge for RTM inversion. UAV-based hyperspectral imaging can capture canopy-scale data but must be calibrated against ground-truth leaf measurements to preserve the physiological relationships we have established. Explicit comparisons between leaf-level and canopy-level models in future field trials will be able to test this scaling directly.

Moreover, the variability in the nutrient availability, pH, and moisture in the soil can indirectly influence nodule development and leaf physiology, confounding spectral signals. For example, heterogeneous soil nitrogen may weaken the link between nodule traits and leaf nitrogen status, reducing model accuracy. While our growth chamber study used uniform substrate and controlled inoculation, field soils contain diverse microbial communities that may interact with the inoculated rhizobia. To improve robustness, field applications of this monitoring approach should integrate soil covariates (e.g., electrical conductivity or organic matter maps) into the predictive models. Hyperspectral data could be fused with soil sensing data to separate soil-induced noise, and spatial stratification during sampling could enhance model generalizability across soil gradients. Advanced validation techniques, such as spatial cross-validation, may also be needed to prevent overfitting to local conditions. Climatic factors, such as diurnal temperature shifts, rainfall, and varying light intensity, can induce transient changes in leaf water content, pigment composition, and stomatal conductance, all of which modulate spectral reflectance. Field drought stress, for instance, is more dynamic than our controlled PEG-6000 treatment and could increase the volatility in nodule weight predictions due to the sensitivity of this trait to water status. Such stressors may also alter the carbon-nitrogen exchange dynamics, affecting the spectral–trait correlations we observed. Longitudinal monitoring, as implemented in our study, will be essential to capture these temporal dynamics. In field settings, time-series hyperspectral data from UAVs could be combined with environmental sensing records to model the climate–nodule interactions. In summary, while our growth chamber research provided a rigorous foundation, deploying this method in the field will require integrated strategies, combining multi-scale level sensing, radiative transfer modeling, soil data fusion, and temporal monitoring, to ensure an accurate and scalable nodule prediction [65].

## CRediT authorship statement

K.H. Cheng: Conceptualization, Methodology, Software, Validation, Formal analysis, Investigation, Data Curation, Writing - Original Draft, Writing - Review & Editing, Visualization, Project administration, Funding acquisition. Kejing Fan: Conceptualization, Methodology, Software, Validation, Formal analysis, Investigation, Data Curation, Writing - Original Draft, Writing - Review & Editing, Visualization, Project administration. Xiewang Gao: Investigation, Writing - Review & Editing. Liping Wang: Investigation, Writing - Review & Editing. Hui Zhang: Investigation, Writing - Review & Editing, Visualization. Feng Zhang: Investigation, Writing - Review & Editing. Fuk-Ling Wong: Investigation, Resources, Writing - Review & Editing. Zhihui Wang: Methodology, Software, Writing - Review & Editing. Jin Wu: Methodology, Software, Resources. Shichao Jin: Writing - Review & Editing, Funding acquisition. Hon-Ming Lam: Conceptualization, Methodology, Validation, Formal analysis, Resources, Data Curation, Writing - Review & Editing, Supervision, Project administration, Funding acquisition.

## Declaration of competing interest

The authors declare the following financial interests/personal relationships which may be considered as potential competing interests: Given his role as Senior Editor, Shichao Jin had no involvement in the peer review of this article and had no access to information regarding its peer review. Full responsibility for the editorial process for this article was delegated to another journal editor.

## Data Availability

The nodulation data and spectral data can be accessed at 10.6084/m9.figshare.31267309.
