# Supplementary material for: Leaf to Root: Harnessing leaf spectral signatures for non-destructive monitoring of soybean nodule traits
Source: Plant Phenomics. 2026 Mar 24;8(2):100203. doi: 10.1016/j.plaphe.2026.100203 (PMC13316468; doi:10.1016/j.plaphe.2026.100203)
Supplement: Multimedia component 1 [file mmc1.docx]

**Table S1.** List of soybean accessions used in this study.

| Accession Name | Type | Origin / Adaptation | Key Characteristics / Notes |
| --- | --- | --- | --- |
| W05 | Wild | Wild soybean | Salt-tolerant |
| W06 | Wild | Wild soybean | Salt-sensitive |
| LH1 | Cultivated | Northern China | Salt-tolerant |
| LH2 | Cultivated | Northern China | Salt-tolerant |
| LH3 | Cultivated | Northern China | Salt-tolerant |
| LH4 | Cultivated | Northern China | Salt-tolerant |
| C12 | Cultivated | Northern China | Salt-tolerant |
| ZH13 | Cultivated | Northern China | Salt-tolerant |
| JD17 | Cultivated | Northern China | Salt-tolerant |
| JHD1 | Cultivated | Northern China | Salt-tolerant |
| LD7 | Cultivated | Northern China | Salt-tolerant |
| SP29 | Cultivated | Northern China | Salt-tolerant |
| HKS-02 | Cultivated | Southern China | Moderately salt-tolerant |
| HKS-03 | Cultivated | Southern China | Moderately salt-tolerant |
| C08 | Cultivated | North America | Moderately salt-tolerant |
| Wm82 | Cultivated | North America | Moderately salt-tolerant |
| Bragg | Cultivated | Australia | Moderately salt-tolerant |
| Bragg-*NARK* | Cultivated | Australia (Mutant) | Supernodulation phenotype; salt-sensitive |

**Table S2.** Formulations of the well-established vegetation indices (VIs) used in this study. R indicates reflectance.

| Index | Formulation | Reference |
| --- | --- | --- |
| CF690 | R690/R600 | Dobrowski et al, 2005 |
| CF740 | R740/R800 | Dobrowski et al, 2005 |
| SR1 | R750/R700 | Gitelson & Merzlyyak, 1997 |
| SR2 | R752/R690 | Gitelson & Merzlyyak, 1997 |
| SR3 | R750/R550 | Gitelson & Merzlyyak, 1997 |
| SR4 | R700/R670 | McMurtey et al. 1994 |
| SRCarter | R760/R695 | Carter, 1994 |
| Gitelson | 1/R700 | Gitelson et al., 1999 |
| Vogelmann1 | R740/R720 | Vogelmann et al., 1993 |
| Vogelmann2 | (R734-R747) - (R715+R726) | Vogelmann et al., 1993 |
| ChlNDI | (R750-R705)/(R750+R705) | Richardson et al, 2002 |
| Double Difference | (R749-R720) - (R701-R672) | le Maire et al., 2004 |
| mSR705 | (R750-R445)/(R705-R445) | Sims & Gamon 2002 |
| Macciioni | (R780-R710)/(R780-R680) | Maccioni et al., 2001 |
| Datt4 | R672/(R550*R708) | Datt, 1998 |
| NDVI | (R850-R650)/(R850+R650) | Robinson & Biehl, 1979 |
| NDWI | (R860-R1240)/(R860+R1240) | Gao, 1996 |
| WI | R900/R970 | Penuelas et al., 1997 |
| PRI | (R531-R570)/(R531+R570) | Gamon et al., 1997 |

**Table S3.** The average nodule numbers of different soybean genotypes under all treatments.

| Lines | Mean Total Nodule Numbers | | | | | | | |
| --- | --- | --- | --- | --- | --- | --- | --- | --- |
|  | Salt2-S | Salt2-B | Salt1-S | Salt1-B | Drought-S | Drought-B | Control-S | Control-B |
| C08 | 47.25^bc^ | 40^ac^ | 43^bc^ | 57.33^ac^ | 37.75^c^ | 51^ac^ | 58^ab^ | 67.25^a^ |
| C12 | 56.25^a^ | 45.5^ab^ | 38.25^ab^ | 34^ab^ | 19.75^b^ | 41.25^ab^ | 44.5^ab^ | 37^ab^ |
| HKS-02 | 19.5^cd^ | 13.75^d^ | 35.25^bd^ | 77.5^a^ | 32.33^bd^ | 45^bc^ | 50.25^b^ | 30.5^bd^ |
| HKS-03 | 53^a^ | 32^a^ | 34.33^a^ | 21^a^ | 36.66^a^ | 40^a^ | 30.5^a^ | 33^a^ |
| JD17 | 75.75^abcd^ | 89.5^abc^ | 61.75^ce^ | 91.5^ab^ | 44.75^e^ | 49.75^de^ | 64.5^be^ | 99.25^a^ |
| JHD1 | 38.5^d^ | 83^bd^ | 59.5^cd^ | 90.5^ab^ | 14.5^cd^ | 60.75^bc^ | 50.5^cd^ | 62^a^ |
| LD7 | 62.75^bc^ | 86.5^ab^ | 88.75^a^ | 75.25^ac^ | 27^e^ | 60^cd^ | 35.25^de^ | 71.5^ac^ |
| LH1 | 26.33^bc^ | 44^ab^ | 39^a^ | 71^ac^ | 39^e^ | 57.5^cd^ | 46.5^de^ | 83.5^ac^ |
| LH2 | 34.5^a^ | 37.75^a^ | 27.5^a^ | 43.75^a^ | 26^a^ | 40.5^a^ | 42.75^a^ | 42.75^a^ |
| LH3 | 26.5^c^ | 52^ab^ | 37.75^bc^ | 65.5^a^ | 33.75^c^ | 51^ab^ | 40.5^bc^ | 52.25^ab^ |
| LH4 | 51^ab^ | 59.75^a^ | 34.75^bc^ | 34.75^bc^ | 35^bc^ | 35.75^bc^ | 43.25^ac^ | 27.75^c^ |
| MU | NA | NA | 330.75^a^ | 201.25^bc^ | 32^d^ | 230.33^ab^ | 104^cd^ | 315.25^a^ |
| SP29 | 89.5^bd^ | 160.25^ab^ | 102.5^bd^ | 210^a^ | 13.5^d^ | 64.75^cd^ | 78.5^bd^ | 142.25^abc^ |
| W05 | 30.75^a^ | 29^a^ | 14.5^b^ | 25^ab^ | 23^ab^ | 26^ab^ | 30.75^a^ | 32.5^a^ |
| W06 | NA | 20^c^ | 33.5^bc^ | 32.75^bc^ | 30.5^bc^ | 41.75^ab^ | 34^bc^ | 54.75^a^ |
| Wm82 | 36.5^cd^ | 51.75^c^ | 58.75^bc^ | 73.75^ab^ | 26^d^ | 94.33^a^ | 42.75^cd^ | 76.25^ab^ |
| Bragg | 85.33^a^ | 53^ab^ | 85.25^a^ | 79^a^ | 11.67^b^ | 10.5^b^ | 25^b^ | 91.25^a^ |
| ZH13 | 68.5^a^ | 61.25^ab^ | 57^ab^ | 71.25^a^ | 53.25^ab^ | 37.5^b^ | 58.5^ab^ | 62.5^ab^ |

MU, Bragg-*NARK* mutant; Salt2, high-concentration (150 mM) salt treatment; Salt1, low-concentration (50 mM) salt treatment; S, CCBAU45436-inoculated; B, USDA110-inoculated. One-way ANOVA with *post hoc* Tukey test was performed for the analysis of statistical significance. Different letters in superscript next to the nodule numbers indicate the corresponding means are significantly different (*P* < 0.05).

**Table S4.** The average nodule weights of different soybean genotypes under all treatments.

| Lines | Mean Total Nodule Weight (g) | | | | | | | |
| --- | --- | --- | --- | --- | --- | --- | --- | --- |
|  | Salt2-S | Salt2-B | Salt1-S | Salt1-B | Drought-S | Drought-B | Control-S | Control-B |
| C08 | 0.2^bc^ | 0.2^ac^ | 0.24^ab^ | 0. 3^a^ | 0.16^c^ | 0.15^c^ | 0.21^bc^ | 0.26^ab^ |
| C12 | 0.21^a^ | 0.27^a^ | 0.18^a^ | 0.15^a^ | 0.18^a^ | 0.11^a^ | 0.22^a^ | 0.21^a^ |
| HKS-02 | 0.09^d^ | 0.14^bd^ | 0.18^abc^ | 0.23^a^ | 0.12^cd^ | 0.13^cd^ | 0.21^ab^ | 0.16^ad^ |
| HKS-03 | 0.22^b^ | 0.14^c^ | 0.21^b^ | 0.19^bc^ | 0.13^c^ | 0. 3^a^ | 0.12^c^ | 0.13^c^ |
| JD17 | 0.13^df^ | 0.22^bc^ | 0.25^ab^ | 0.2^bd^ | 0.11^ef^ | 0.09^f^ | 0.3^a^ | 0.17^cde^ |
| JHD1 | 0.15^bcd^ | 0.18^ac^ | 0.2^ac^ | 0.23^ab^ | 0.05^e^ | 0.08^de^ | 0.26^a^ | 0.12^ce^ |
| LD7 | 0.27^a^ | 0.36^a^ | 0.34^a^ | 0.28^a^ | 0.08^b^ | 0.12^b^ | 0.25^a^ | 0.14^b^ |
| LH1 | 0.12^c^ | 0.24^ab^ | 0.19^bc^ | 0.28^a^ | 0.12^c^ | 0.18^bc^ | 0.18^bc^ | 0.32^a^ |
| LH2 | 0.14^bc^ | 0.16^bc^ | 0.18^ac^ | 0.23^a^ | 0.12^c^ | 0.12^c^ | 0.19^ab^ | 0.17^ac^ |
| LH3 | 0.18^ac^ | 0.25^a^ | 0.16^ac^ | 0.24^ab^ | 0.12^c^ | 0.13^c^ | 0.2^ac^ | 0.15^bc^ |
| LH4 | 0.22^a^ | 0.21^a^ | 0.17^a^ | 0.16^a^ | 0.21^a^ | 0.14^a^ | 0.19^a^ | 0.12^a^ |
| MU | NA | NA | 0.49^a^ | 0.34^b^ | 0.06^c^ | 0.23^b^ | 0.32^b^ | 0.28^b^ |
| SP29 | 0.25^cd^ | 0.35^abc^ | 0.45^ab^ | 0.47^a^ | 0.11^d^ | 0.15^d^ | 0.37^abc^ | 0.27^bd^ |
| W05 | 0.13^ab^ | 0.15^a^ | 0.08^ab^ | 0.12^ab^ | 0.06^b^ | 0.12^ab^ | 0.08^ab^ | 0. 1^ab^ |
| W06 | NA | 0.12^ac^ | 0.11^bc^ | 0.18^a^ | 0.1^c^ | 0.13^ac^ | 0.13^ac^ | 0.16^ab^ |
| Wm82 | 0.16^d^ | 0.31^ab^ | 0.22^cd^ | 0.28^bc^ | 0.14^d^ | 0.38^a^ | 0.21^cd^ | 0.28^bc^ |
| Bragg | 0.13^ac^ | 0.09^bc^ | 0.17^ab^ | 0.19^a^ | 0.1^bc^ | 0.04^c^ | 0.21^a^ | 0.11^bc^ |
| ZH13 | 0.34^a^ | 0.27^b^ | 0.18^de^ | 0.24^bc^ | 0.13^e^ | 0.16^de^ | 0.21^cd^ | 0.15^e^ |

MU, Bragg-*NARK* mutant; Salt2, high-concentration salt treatment (150 mM); Salt1, low-concentration (50 mM) salt treatment; S, CCBAU45436-inoculated; B, USDA110-inoculated. One-way ANOVA with *post hoc* Tukey test was performed for the analysis of statistical significance. Different letters in superscript next to the nodule numbers indicate the corresponding means are significantly different (*P* < 0.05).

**Table S5.** Partial Least Squares Regression (PLSR) model performance for the estimation of nodule number and nodule weight of *Rhizobium* strains (USDA110 or CCBAU45436) and abiotic stress (drought or salt).

|  | N | Nodule number | | | Nodule weight | | |
| --- | --- | --- | --- | --- | --- | --- | --- |
|  |  | R^2^ | RMSE | nRMSE | R^2^ | RMSE | nRMSE |
| CCBAU45436 | 266 | 0.59 | 27.16 | 6.72% | 0.61 | 0.057 | 11.40% |
| USDA110 | 262 | 0.57 | 34.81 | 8.70% | 0.42 | 0.068 | 14.05% |
| Salt | 255 | 0.68 | 28.95 | 7.17% | 0.60 | 0.057 | 12.10% |
| Drought | 132 | 0.57 | 22.15 | 8.36% | 0.11 | 0.066 | 14.62% |
| Control | 141 | 0.35 | 42.65 | 10.82% | 0.25 | 0.072 | 17.35% |

RMSE, Root Mean Square Error; nRMSE, normalized RMSE.

**Table S6**. Optimal spectral domains generated by the Partial Least Squares Regression (PLSR) model for nodule number (NN) and total nodule weight (NW) across distinct *Rhizobium* strains (CCBAU45436 or USDA110) and across abiotic stress (salt or drought).

|  | NN | NW |
| --- | --- | --- |
| CCBAU45436 | 400-423 nm, 520-560 nm, 673-772 nm, 987-1025 nm, 1117-1162 nm, 1290-1405 nm, 1850-1915 nm | 400-455 nm, 500-550 nm, 660-750 nm, 985-1025 nm, 1120-1162 nm, 1295-1415 nm, 1683-1723 nm, 1850-1925 nm |
| USDA110 | 400-460 nm, 530-575 nm, 665-752 nm, 1120-1160 nm, 1290-1410 nm, 1860-1910 nm. | 400-475 nm, 675-745 nm, 962-988 nm, 1000-1040 nm, 1113-1166 nm, 1286-1414 nm, 1853-1925 nm |
| Salt | 400-431 nm, 526-546 nm, 670-760 nm, 984-1017 nm, 1117-1166 nm, 1286-1402 nm, 1860-1908 nm | 400-464 nm, 517-548 nm, 672-753 nm, 975-1030 nm, 1120-1166 nm, 1290-1415 nm, 1865-1919 nm |
| Drought | 640-708 nm, 715-728 nm, 1863-1908 nm | 400-422 nm, 655-708 nm, 710-756 nm, 1305-1410 nm, 1857-1909 nm |

**
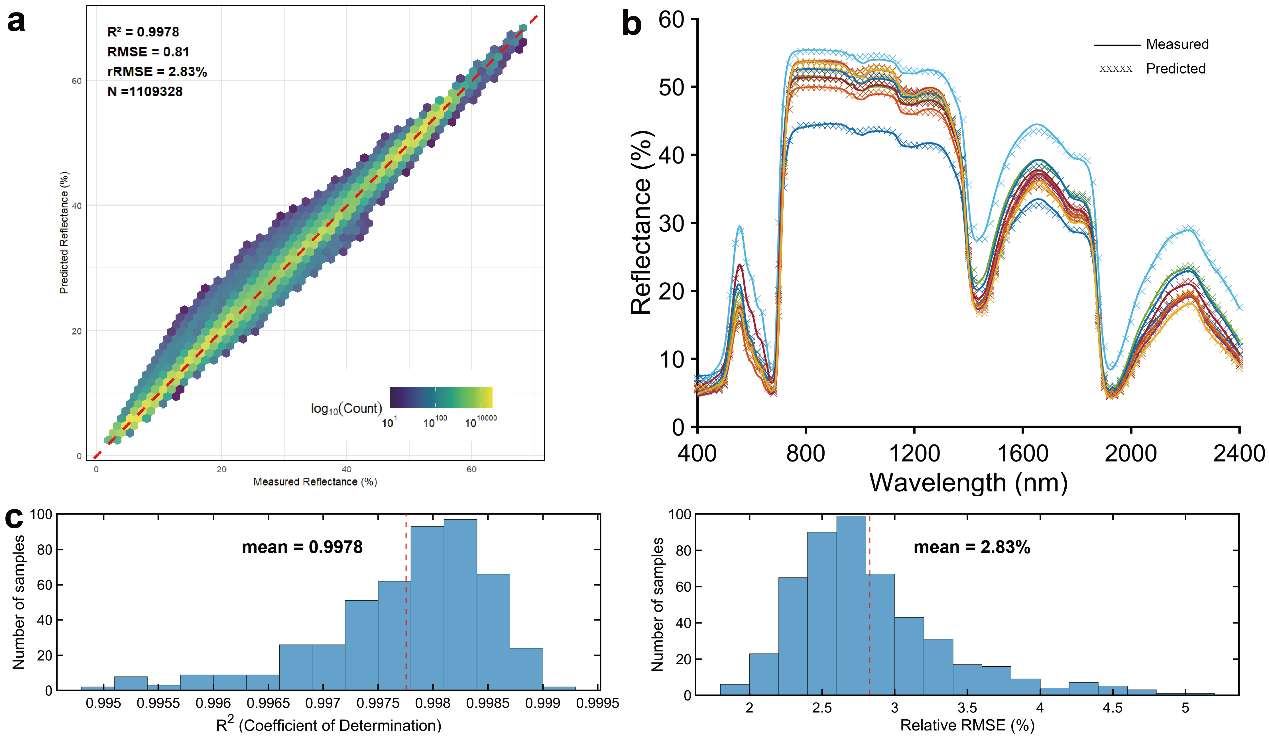
Fig. S1.** Evaluating the PROSPECT model inversion accuracy. The PROSPECT-5 model inverted the leaf reflectance spectra to estimate biophysical traits by optimizing the parameters within physiologically plausible ranges. The prior ranges for the key parameters were set as follows, based on the typical values for soybean leaves and published literature (Zhong et al., 2025): chlorophyll a+b content (Cab): 0–140 μg cm^-^²; carotenoid content (Car): 0–50 μg cm^-^²; anthocyanin content (Anth): 0–10 mg cm^-^² (or equivalent units); brown pigment content (Cbrown): 0–5 (unit-less, representing senescent material); equivalent water thickness (EWT): 0.000–0.1 cm. These ranges were chosen to encompass the expected variations among soybean leaves under controlled stress conditions, ensuring that the optimization algorithm searched within biologically realistic bounds. The inversion was implemented in MATLAB R2024a with the trust-region reflective algorithm. The specific settings were: maximum iterations: 400; maximum function evaluations: 1,000; convergence threshold: 1×10^-6^; function tolerance: 1×10^-6^. These settings ensured efficient convergence while minimizing the risk of overfitting. The PROSPECT model was validated using three visualization methods. (a) A hexbin density plot comparing the predicted versus measured reflectance values and calculated key performance metrics (R² = 0.9978, RMSE = 0.81, relative RMSE = 2.83%). RMSE, root mean square error. (b) The representative spectra (400 – 2,500 nm) from six randomly selected samples were displayed, each represented by a different color, with measured reflectance in solid lines and the corresponding model predictions marked by X’s at 100-nm intervals. (c) The distributions of R² and relative RMSE values from each sample were plotted as histograms to assess the model consistency across samples. The red dotted lines represent the mean value of R^2^ and relative RMSE, respectively.


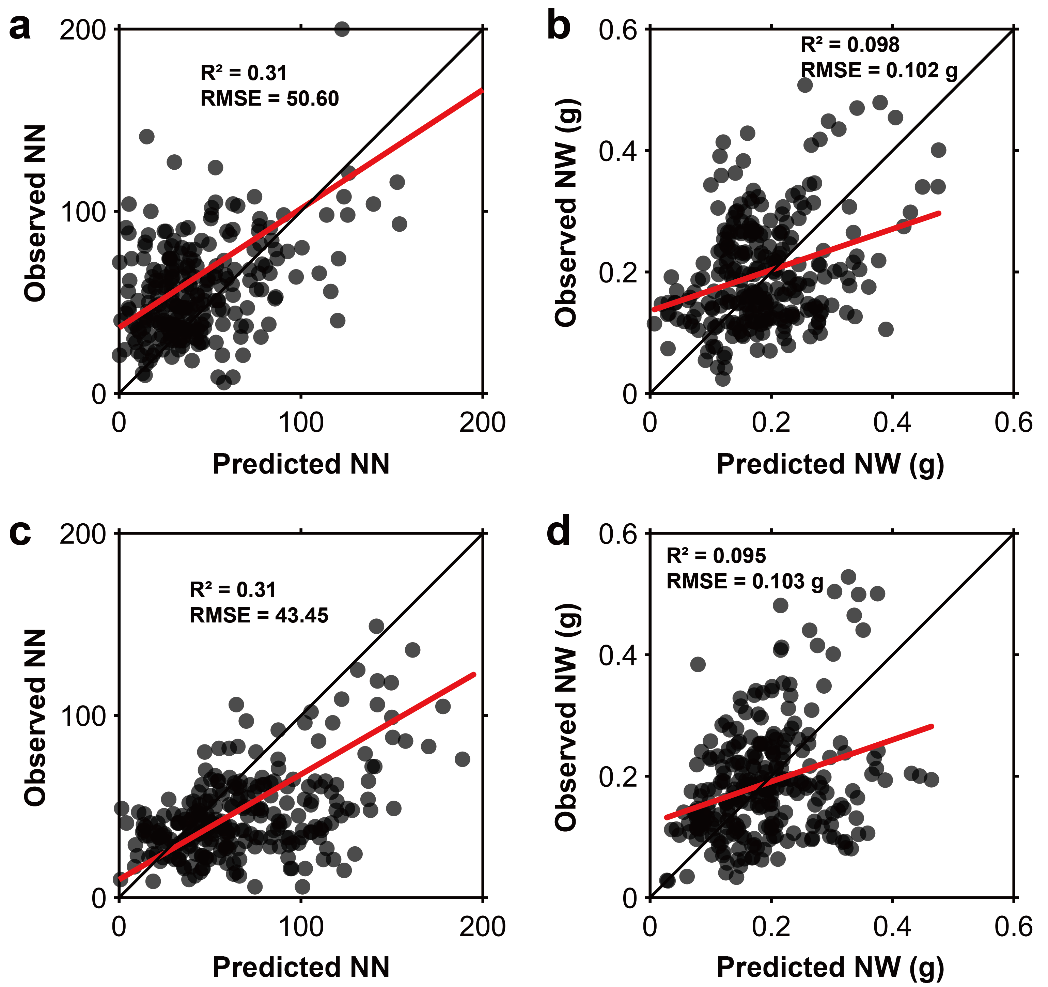


**Fig. S2.** Model cross validation for distinct *Rhizobium* strains (USDA110 and CCBAU45436). (a) Predicting nodule number (NN) for the USDA110-inoculated dataset with the CCBAU45436-derived PLSR model. (b) Predicting nodule weight (NW) for the USDA110-inoculated dataset with the CCBAU45436-derived PLSR model. (c) Predicting NN for the CCBAU45436-inoculated dataset with the USDA110-derived PLSR model. (d) Predicting NW for the CCBAU45436-inoculated dataset with the USDA110-derived PLSR model.


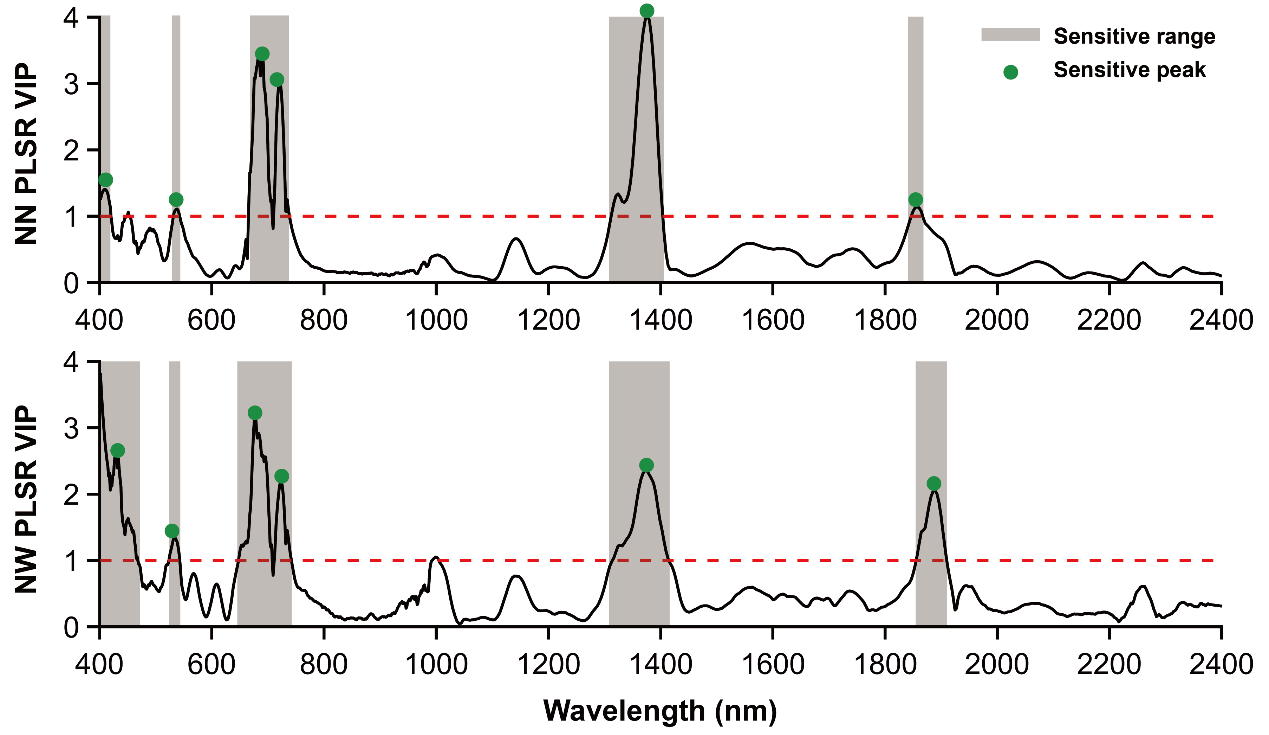


**Fig. S3.** Variable importance projection (VIP) of the Partial Least Squares Regression (PLSR) model for the control group (N = 141). The red dotted line in each panel indicates a VIP value of 1. The spectral regions above the dotted lines were identified as significant.

**References**

Carter, G. A. (1994). Ratios of leaf reflectances in narrow wavebands as indicators of plant stress. *Remote sensing*, *15*(3), 697-703.

Datt, B. (1998). Remote sensing of chlorophyll a, chlorophyll b, chlorophyll a+ b, and total carotenoid content in eucalyptus leaves. *Remote sensing of environment*, *66*(2), 111-121.

Dobrowski, S. Z., Pushnik, J. C., Zarco-Tejada, P. J., & Ustin, S. L. (2005). Simple reflectance indices track heat and water stress-induced changes in steady-state chlorophyll fluorescence at the canopy scale. *Remote sensing of environment*, *97*(3), 403-414.

Gamon, J., Serrano, L., & Surfus, J. S. (1997). The photochemical reflectance index: an optical indicator of photosynthetic radiation use efficiency across species, functional types, and nutrient levels. *Oecologia*, *112*(4), 492-501.

Gao, B. C. (1996). NDWI—A normalized difference water index for remote sensing of vegetation liquid water from space. *Remote sensing of environment*, *58*(3), 257-266.

Gitelson, A. A., & Merzlyak, M. N. (1997). Remote estimation of chlorophyll content in higher plant leaves. *International journal of remote sensing*, *18*(12), 2691-2697.

Gitelson, A. A., Buschmann, C., & Lichtenthaler, H. K. (1999). The chlorophyll fluorescence ratio F735/F700 as an accurate measure of the chlorophyll content in plants. *Remote sensing of environment*, *69*(3), 296-302.

Le Maire, G., François, C., & Dufrene, E. (2004). Towards universal broad leaf chlorophyll indices using PROSPECT simulated database and hyperspectral reflectance measurements. *Remote sensing of environment*, *89*(1), 1-28.

Maccioni, A., Agati, G., & Mazzinghi, P. (2001). New vegetation indices for remote measurement of chlorophylls based on leaf directional reflectance spectra. *Journal of Photochemistry and Photobiology B: Biology*, *61*(1-2), 52-61.

McMurtrey Iii, J. E., Chappelle, E. W., Kim, M. S., Meisinger, J. J., & Corp, L. A. (1994). Distinguishing nitrogen fertilization levels in field corn (Zea mays L.) with actively induced fluorescence and passive reflectance measurements. *Remote sensing of environment*, *47*(1), 36-44.

Penuelas, J., Pinol, J., Ogaya, R., & Filella, I. (1997). Estimation of plant water concentration by the reflectance water index WI (R900/R970). *International journal of remote sensing*, *18*(13), 2869-2875.

Richardson, A. D., Duigan, S. P., & Berlyn, G. P. (2002). An evaluation of noninvasive methods to estimate foliar chlorophyll content. *New phytologist*, *153*(1), 185-194.

Robinson, B., & Biehl, L. L. (1979). Calibration procedures for measurement of reflectance factor in remote sensing field research. In *Measurements of optical radiations* (Vol. 196, pp. 16-26). SPIE.

Sims, D. A., & Gamon, J. A. (2002). Relationships between leaf pigment content and spectral reflectance across a wide range of species, leaf structures and developmental stages. *Remote sensing of environment*, *81*(2-3), 337-354.

Vogelmann, J. E., Rock, B. N., & Moss, D. M. (1993). Red edge spectral measurements from sugar maple leaves. *TitleREMOTE SENSING*, *14*(8), 1563-1575.

Zhong, W., Cheng, K. H., Zhou, L., Zhang, J., Zhang, F., Feng, G. & Wu, J. (2025). Metabolite-derived spectral modelling can differentiate heat and drought stress under hot-dry environments. Remote Sensing of Environment, 328, 114884.
